# Optimization and Validation of Maceration-Mediated Hydrodistillation to Extract Caryophyllene-Rich Essential Oil from Sea Buckthorn Berries

**DOI:** 10.17113/ftb.63.01.25.8607

**Published:** 2025-03

**Authors:** Zainab Liaqat, Sumia Akram, Rizwan Ashraf, Muhammad Umair Kamal, Rabia Naeem, Muhammad Mushtaq

**Affiliations:** 1 Department of Chemistry, Government College University Lahore, Lahore 54000, Punjab, Pakistan; 2 Division of Science and Technology, University of Education Lahore, Lahore 54770, Punjab, Pakistan; 3 Department of Chemistry, University of Agriculture, Faisalabad, Faisalabad 38000, Punjab, Pakistan

**Keywords:** sea buckthorn berries, essential oil, caryophyllene, maceration-mediated hydrodistillation, GC-MS, antioxidant activity

## Abstract

**Research background:**

Hydrodistillation is a convenient and economical method to extract essential oils, but this technique has been abandoned due to limited extraction rates. Comparison to conventional hydrodistillation, maceration-mediated hydrodistillation could increase mass transfer and provide better control over the extraction thermodynamics, thereby preserving the aroma constituents and their antioxidant activities. The present study describes a useful and innovative modification of conventional hydrodistillation by introducing a macerating agent Triton X-100 and NaCl as an electrolyte to accelerate mass transfer for better extraction of caryophyllene-rich essential oil from sea buckthorn berries.

**Experimental approach:**

The parameters of maceration-mediated hydrodistillation, including the mass fraction of macerating agent, electrolyte concentration and extraction time, were investigated within a wide range of 1−10 %, 1−10 g/100 mL and 3−8 h, respectively, to increase the oil yield (g/100 g). The parameters were optimized according to the desirability approach using response surface methodology. The antioxidant activity of the essential oil obtained under optimal conditions was measured using *in vitro* antioxidant assays and its aroma profile using gas chromatography with mass spectrometery (GC-MS).

**Results and conclusions:**

The optimized parameters for the modified hydrodistillation were observed at 4.22 mL Triton X-100 and 4.03 g NaCl for 5.61 h of extraction time with the essential oil yield of (3.2±0.1) % compared to 2.1 % obtained with conventional hydrodistillation. The essential oil produced by the assisted hydrodistillation was rich in (−)-β-caryophyllene (37.2 %) with good antioxidant activities in terms of free radical scavenging capacity (84.2 %), inhibition of linoleic acid peroxidation (68.2 %) and antioxidant capacity expressed in Trolox equivalents (168 µmol/mL).

**Novelty and scientific contribution:**

Triton X-100 can disrupt the cell membrane to release the bioactive compounds, while the NaCl reduces the solubility of the non-polar components of the essential oil in the aqueous phase, which can ultimately improve the extraction yield. The proposed approach can be used with minor modifications with the existing hydrodistillation setups and it seems to be more economical for the extraction of sea buckthorn essential oil without compromising its antioxidant potential or its valuable aroma compounds on an industrial scale.

## INTRODUCTION

The sea buckthorn (*Hippophae rhamnoides* L.), also known as sea berry and Siberian pineapple, is an exceptionally important shrub of the Elaeagnaceae family (*1*). It grows worldwide, but it is mainly distributed as a wild shrub in the temperate zones of Pakistan, China and northern Afghanistan (*2*). In recent decades, this shrub has attracted the attention of medicinal chemists due to its interesting phytochemical profile and medicinal importance (*3*). The essential oil of sea buckthorn is rich in terpenoids, palmitoleic acid, oleic acid and tocopherols (*4*) and is used in cosmetic, food and pharmaceutical industries (*5*) due to its interesting pharmacological properties (*2*). Numerous reports claim that sea buckthorn essential oil is a promising solution for the treatment of depression (*6*), a hypolipidaemic agent as well as an anti-inflammatory agent as it contains caryophyllene (*1*, *7*).

There has been a meteoric rise in the consumption of natural essential oils and flavouring agents in food, cosmetic and pharmaceutical industries, which requires a high quality and abundant supply. Therefore, the extraction of the essential oil by conventional methods like hydrodistillation is becoming a challenge as it is difficult to meet the market demand due to poor efficiency and compromised quality (*8*, *9*). A number of alternative approaches have been put into practice: (*i*) ultrasound-assisted extraction (*10*), (*ii*) microwave and thermal extraction (*11*), (*iii*) supercritical fluid extraction (*12*-*14*), subcritical (*15*) and organic solvent extractions (*4*), and (*iv*) enzyme-assisted extraction (*16*). Although these approaches improved the extraction yields, they face challenges related to cost and complexity of the distillation of essential oils.

Hydrodistillation of essential oils is an expedient, inexpensive and sustainable method, but it is not an option due to poor extraction efficiency mainly because of limited mass transfer and heat transfer rates (*17*). It has been observed that the addition of surfactants or macerating agents can accelerate the mass transfer (*18*). Similarly, the presence of electrolytes or salt in the extraction media can help us gain more control over the mass transfer rate and thermodynamics. Therefore, we planned to introduce the use of macerating agents (non-ionic surfactants) and salt for the improved extraction of sea buckthorn essential oil. The essential oil extracted under optimum modified hydrodistillation conditions was further analysed by gas chromatography coupled with mass spectrometry (GC-MS) and *in vitro* antioxidant assays in aqueous and organic asolvents to determine its antioxidant properties and main constituents, respectively.

## MATERIALS AND METHODS

### Preparation of sea buckthorn berries

The sea buckthorn berries were collected from a local supplier (Akhter Corporation, Karachi, Pakistan), dried under vacuum until no further mass loss was observed and ground into coarse particles using a household grinder AG-639 Deluxe (Anex Electrical Co Ltd, Hong Kong, SAR China). The ground berries were sieved and stored in a zipper bag for further use.

### Procurement of supplies

The Clevenger tube having 250-mm collector and 175-mm fractionating column was prepared in a local glass-blowing workshop. The Sigma-Aldrich Chemie, Merck (GmbH, Darmstadt, Germany) supplied all the standards and reagents: ABTS (di-ammonium salt of 2,2-azinobis(3-ethylbenzothiazoline-6-sulphonic acid), DPPH (2,2-diphenyl-1-picrylhydrazyl), Trolox (6-hydroxy-2,5,7,8-tetramethylchromane-2-carboxylic acid), gallic acid, linoleic acid and BHT (butylated hydroxytoluene). The Triton X-100 and anhydrous sodium sulfate (ethereal solution drying agent) were procured from Duksan (Ansan, South Korea). The analytical grade solvents and chemicals such as petroleum ether, methanol, ethanol, sodium phosphate buffer, Fe(II) chloride, NaCl and ammonium thiocyanate were supplied by Merck (Darmstadt, Germany).

### Extraction of sea buckthorn essential oil

For the extraction of the essential oil, 100 g of precisely weighed sea buckthorn berry powder were transferred to a round bottom flask already filled with 250 mL of double distilled water containing a specific amount of salt and the macerating agent according to the conditions given in Table 1. The flask was connected to the Clevenger apparatus (*19*) with different side arm lengths and dimensions to study the effects of tube design and length. The entire set up was connected to a condenser set at (10±2) °C by circulating a coolant through it. While conducting all experiments listed in Table 1, the temperature of the extraction mixture (round bottom flask) was kept at 105 °C, while pressure was kept at 101325 Pa by leaving the top of the Clevenger tube open. Finally, the effect of extraction time, amounts of macerating agent (Triton X-100) and NaCl was evaluated at five different levels (Table 1). The amount of essential oil accumulated in the collector was transferred to pre-weighed bottles of ethereal solution and weighed again to calculate the yield according to the following equation:


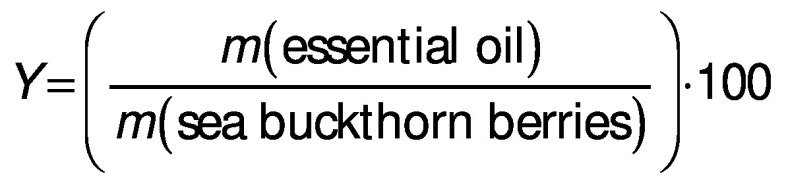
 /1/

**Table 1:** The experimental conditions for the maceration-mediated hydrodistillation of the essential oil from sea buckthorn berries

Factor	Central composite experimental design point				
	-α	-1	0	1	α
A: *V*(Triton X-100)/*m*(sea buckthorn)/(mL/100 g)	1	2.82	5.5	8.18	10
B: *m*(NaCl)/g	1	2.82	5.5	8.18	10
C: *t*/h	5	5.61	6.5	7.39	8

*(+α and –α), (+1 and −1), and (0) represent axial, factorial and centre points of the proposed experimental design

The organic layer was subsequently mixed with 5 g of anhydrous sodium sulfate to remove any moisture and stored under darkness ((10±2) °C) for further use.

### Experimental layout

The preliminary screening experiments showed that surfactant concentration, salt content and extraction time affect the extraction of aroma compounds from sea buckthorn berries. Based on these observations, the surfactant ratio (A), salt content (B) and extraction time (C) were applied at five different levels, coded as *α*, 1, 0, -1 and -*α* (Table 1) in a central composite design in the response surface methodology. A fully rotatable approach was followed, with a total of 20 runs, including six replicates, the centre point (coded as 0), eight runs for the axial point (coded as +α and -α) and six runs for factorial points (coded as +1 and -1).

### Chromatographic analysis of essential oil

The individual components of the essential oil produced under optimal conditions were analysed using gas chromatography with quadrupole mass spectrometry (GC-MS). The analysis was carried out using Shimadzu GCMS-QP2010 (Kyoto, Japan) equipped with a 30 m×0.25 mm capillary column (SH-Rxi®-5Sil MS; Shimadzu) made of cross-linked 1,4-bis(diphenyl/dimethyl) polysiloxane (5 % diphenyl/95 % dimethyl polysiloxane) stationary phase with a particle size of 0.25 µm. The analysis conditions include the use of helium (99.99 %) as the carrier gas. The flow rate, linear velocity and purge flow were set to 1.5 mL/min, 55.5 cm/s and 0.0 mL/min, respectively. The column oven temperature was programmed with a hold time of 3.0 min at the start (60.0 °C) and end point (300 °C), with a ramp rate of 6.0 °C. A volume of 3.0 µL of essential oil prepared using modified hydrodistillation and conventional hydrodistillation was injected manually at an injection volume of 3.0 µL with split-type injection mode at a pressure of 150 kPa and a split ratio of 80/20. The constituents of the essential oil were detected and quantified using QP-MS under total ion collector (TIC) mode and scanning from 40 to 500 *m*/*z*. The QP-MS operating parameters, namely ion source temperature, interface temperature and solvent cut-off time were set at 150 and 300 °C and 2.5 min, respectively. After analysis, the obtained mass fragmentations of each compound were compared with the National Institute of Standards and Technology (NIST) Library provided by Shimadzu (Kyoto, Japan) and the literature to authenticate the compounds present in the essential oil.

### Antioxidant properties of essential oil

The antioxidant capacity of essential oil extracted by thermodynamically modified hydrodistillation and conventional hydrodistillation was monitored by measuring their ability to scavenge free radicals (*20*), inhibit linoleic acid peroxidation (*15*) and antioxidant capacity expressed in Trolox equivalents (*21*).

#### Free radical scavenging capacity

The ability of the essential oil produced using modified hydrodistillation and conventional hydrodistillation to scavenge 1,1-diphenyl 2-picrylhydrazyl free radicals (DPPH˙) was determined by simply incubating the essential oil with DPPH˙ (*20*). Briefly, 500 µL of the essential oil was mixed with 500 µL of freshly prepared 1.0 mg/L solution of DPPH˙ in HPLC grade methanol and kept in the dark for 15 min. The absorbance of the incubated solution (*A*_s_) was measured at 517 nm against the original DPPH solution, which served as a control (*A*_c_) using a microplate reader (ELX 800; Bioteck, Winooski, VT, USA) to calculate the inhibition of DPPH by essential oil:


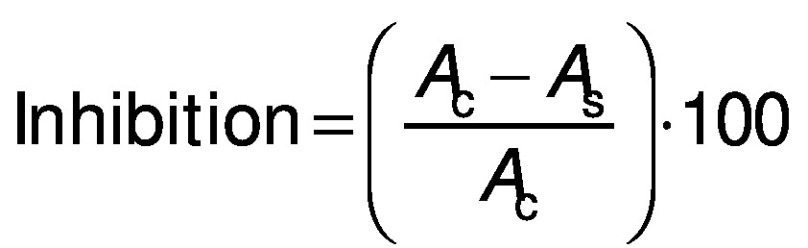
 /2/

#### Inhibition of linoleic acid peroxidation

The ability of the essential oil to retard the formation of peroxides in linoleic acid was investigated according to the method described by Zheng *et al.* (*15*). The assay was slightly modified. Briefly, 500 µL of essential oil of both samples, obtained by modified hydrodistillation or conventional hydrodistillation were added to a mixture containing 100 µL of 10 % linoleic acid, followed by the addition of 500 µL of 0.2 M phosphate buffer (pH=7.0) and 750 µL of ethanol. The linoleic acid in the resulting mixture was oxidised at 40 °C for 72 h. At the end of incubation, 200 µL of the above mixture were further diluted with 500 µL of ethanol, treated with 30 % ammonium thiocyanate (1.0 mL) and 200 µL of 20 mM FeCl_2_ in HCl (3.5 %) and the obtained mixture was incubated at 40 °C for 3 min. The amount of peroxides formed in the solution was determined by estimating the formation of thiocyanates, *i.e.* by measuring the absorbance (*A*_s_) at 500 nm using phosphate buffer as control (*A*_c_) according to Eq. 2.

#### Trolox equivalent antioxidant capacity

The Trolox equivalent antioxidant capacity (TEAC) of essential oil obtained by both methods was evaluated according to the TEAC assay as reported by Mushtaq *et al.* (*21*). The ABTS radical cations were prepared by treating 100 mL of 7.0 mM diammonium salt of ABTS (2,2-azinobis(3-ethylbenzothiazoline-6-sulfonic acid)) with 50 mL of 2.45 mM potassium hydrogen sulphate in the dark at ambient temperature for 8.0 h. The resulting ABTS˙^+^ was diluted in ethanol until the absorbance of the solution dropped to 0.70±0.05 at 734 nm (*λ*_max_). Equal volumes (100 µL) of the essential oil and ABTS^·+^ were then mixed in a 96-well plate, incubated for another 8 min and read at the above wavelength to calculate the percentage of scavenging of ABTS (Eq. 2) by the essential oil (*A*_s_). The synthetic antioxidant Trolox was used as a positive control (*A*_c_) to express the antioxidant capacity and the results are expressed as Trolox equivalents per mL of essential oil.

### Statistical analysis and optimization

The optimized yield of essential oil obtained by two methods was statistically analysed by analysis of variance (ANOVA) of a statistical workstation Design Expert, v. 12.0.0.3.0 (*22*). Experimental layout comprising six replicates was made at the centre points to calculate mean square error (MSE) as well as each treatment (axial or factorial point) that had a significant (p≤0.05) effect on the yield was modulated according to the regression equation:


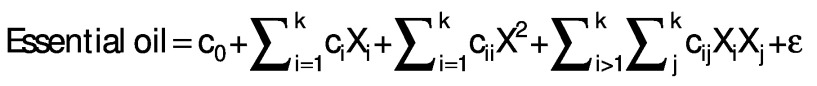
 /3/

where c_0_ and *ε* are noise (intercept) and pure error, while X_i_, X^2^ and X_i_X_j_ are linear, quadratic and interaction effects of the conditions for modified hydrodistillation. The chi-squared test (χ^2^ (1, N=182)≥3.84) was used to measure any significant (p≤0.05) differences between treatments or variables. A p≤0.01 was also used to test the fitness of the model and the agreement between observed and predicted values.

All factors significantly (p≤0.05) affecting the extraction of the essential oil were further transformed into the so-called desirability (d) considering the relative importance of the factor, and the conditions with the highest values of d were validated in a separate set of triplicate experiments.

## RESULTS AND DISCUSSION

### Preliminary screening of hydrodistillation conditions

In a preliminary screening of hydrodistillation experiments, it was found that increasing water temperature increased the recovery rates, but it also increased the water content in the collected essential oil. On the other hand, significant extraction of essential oil from sea buckthorn berries could not be achieved by lowering the temperature below 60 °C. This finidng is supported by literature reports on experimental conditions of hydrodistillation in laboratories that maintained temperature of the extraction solvent close to the boiling point of water. However, the addition of a non-volatile electrolyte can increase the boiling point of water and thus higher temperature could improve extraction rates.

The size of the Clevenger tube also affected the extraction of the essential oil. The analysis of the experimental results with different side arm lengths, angles (bending) and vertical column heights of Clevenger tubes shows that Clevenger tube with a 150 mm long column and 175 mm bent tube (at an angle of 120°) can provide a better essential oil yield. Besides, the physicochemical properties of the water, especially the heat capacity and polarity, need to be optimized. Interestingly, small amounts (about 10 %) of Triton X-100 and NaCl acted as activators by influencing the colligative properties of the extraction solvent that resulted in improved extraction of the essential oil. This increase in extraction due to the addition of activators could be related to the maceration and micellisation potential of the surfactant and the change in the heat capacities of the extraction solvents. It has already been observed that Triton X-100 disrupts cell membranes to release the bioactive compounds by lowering the surface tension of water (*18*, *23*, *24*). Meanwhile, NaCl affects the formation of a suspension between oil and water to reduce the solubility of non-polar components of the essential oil in the aqueous phase and alter the cell wall structure, which ultimately improves the extraction yield (*24*-*27*). The addition of these activators was further investigated in five different amounts to optimize the extraction yield.

### Optimisation of modified hydrodistillation

The yield of the essential oil obtained by modified hydrodistillation under different extraction parameters was investigated and optimized over a range of non-ionic surfactant volumes (A), NaCl mass (B) and extraction time (C) using the rotatable central composite design (Table 1). The analysis of variance (Table S1) of the essential oil (*w*(sea buckthorn berry)=g/100 g) showed that the selected factors affected the extraction of the essential oil. Overall, the linear (A, B and C), quadratic (A^2^, B^2^ and C^2^) and interaction (AB and AC) effect of all these parameters on the recovery of essential oil from sea buckthorn was significant (p≤0.05) except for the BC interaction with p>0.05. Also, the high F-value (795.54) of the model indicates that the model is significant and that there is only 0.01 % variation due to noise.

The change in the yield of essential oil from sea buckthorn in response to the addition of non-ionic surfactant (Triton X-100) and NaCl could be due to a change in the microstructure of the plant (maceration) and the heat capacity of the water (*24*). In addition, Triton X-100 can solubilise the lipid bilayer by reducing the surface tension between the two phases, thereby increasing mass transfer. However, the increase in surfactant concentration led to the formation of micelles that entrapped the oil droplets. In this scenario, NaCl increased the diffusivity of the solvent, allowing it to penetrate the micelles and contribute to the release of the essential oil. The amounts of Triton X-100 and NaCl must be carefully controlled, as increasing NaCl or Triton X-100 above 5-6 % of the sample may negatively affect the essential oil yield. This effect on the yield may be due to the higher boiling point of the extraction medium by the addition of salt, which improves heat and mass transfer. In this way, the presence of NaCl or another electrolyte can provide thermodynamic control over the extraction process, ultimately making the process faster and more economical. Hydrodistillation with water, which has a high boiling point, can also extract the components with boiling points higher than that of water (*24*). The extraction time is the third important factor affecting the extraction of essential oil from plant material (*28*).

**Fig. 1:**
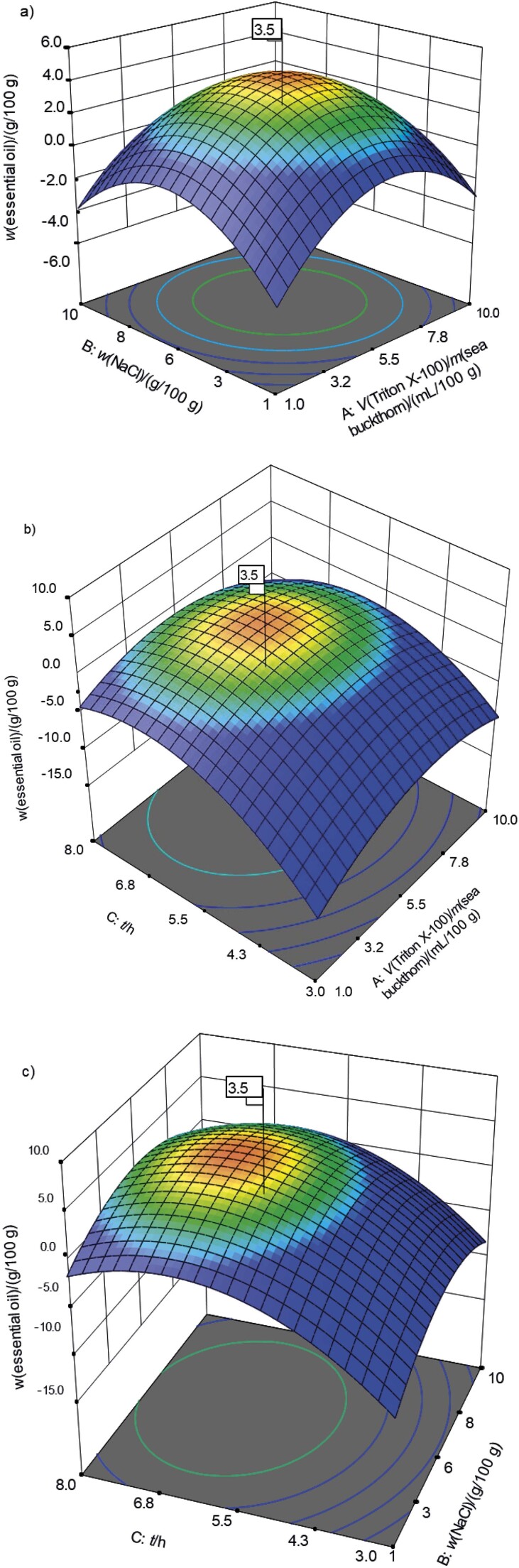
Three-dimensional surface plots of the interaction between different extraction parameters: a) Triton X-100 *vs* NaCl, b) Triton X-100 *vs* time, and c) NaCl *vs* time, which influence the recovery of the essential oil from sea buckthorn

**Fig. S1:**
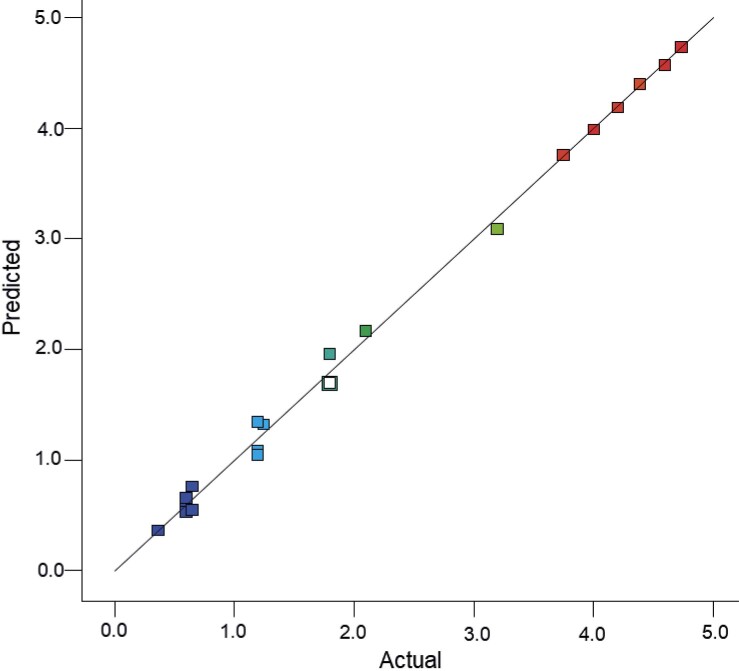
Comparison between predicted and actual values of the essential oil from sea buckthorn

The interactions between these three parameters (AB, AC and BC) can be better understood from the data shown in Fig. 1. The results in Fig. 1a and Fig. 1b show that increasing the volume of Triton X-100 up to 5.5 mL increases the yield of essential oil, while adding more Triton X-100 can negatively affect the yield of essential oil. However, the addition of NaCl together with Triton X-100 (5.5-7.7 mL) improved the essential oil yield. These factors were optimized using a statistical model to improve the yield and make the process more economical and rapid (*23*). The quadratic effect of all extraction parameters (A^2^, B^2^ and C^2^) also had a significant effect on the essential oil yield (Table S1). Moreover, the coefficient of determination (R^2^) value of 0.9986 confirms the agreement between observed and predicted values, and the values for adjusted and predicted R^2^, *i.e.* 0.9974 and 0.9930, respectively, indicate the absence of outliers. A coefficient of variation of 4 % confirms the validity and reliability of the observed data. Finally, Fig. S1 shows visual evidence of the agreement between the observed and predicted yield of essential oil. Overall, the yield of essential oil can be modulated with the following equation:


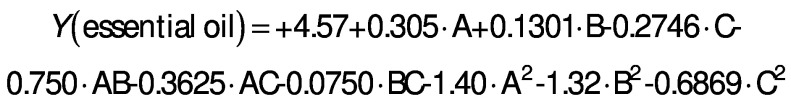
 /4/

### Validation of optimal extraction parameters

To test the effect of different thermodynamically modified maceration parameters on the yield of essential oil, the amounts of Triton X-100 (2.8−10.0 mL), NaCl (2.8−10.0 g) and extraction time (5−8 h) were investigated. Sea buckthorn berries processed under the above conditions were found to have a higher essential oil yield than those treated by conventional hydrodistillation. The statistical predictions indicate that the modified hydrodistillation can recover about 3.1 % essential oil under the optimal extraction conditions, *i.e.* 4.22 mL Triton X-100, 4.03 g NaCl and 5.61 h of extraction time. The suitability of the model equations for predicting the response values was confirmed by performing a validation experiment under these optimized conditions, under which (3.2±0.1) g/100 g of essential oil were obtained (Table 2 (*29*-*31*)). It is clear from the validation experiments that the predicted optimal conditions allow higher yield of essential oil. A careful review of the previously published research shows that in no study was the plant material macerated and the thermodynamics of the water modified simultaneously, as is the case in the present study. However, Cakir (*29*) extracted the essential oil of sea buckthorn by conventional steam distillation with a yield of 0.1 % (*m*/*m*), which consists mainly of alcohols, terpenes, aliphatic esters and hydrocarbons. Yue *et al.* (*30*) obtained 0.03, 1.26 and 0.37 % essential oil from sea buckthorn seeds, pulp and leaves, respectively, by conventional hydrodistillation. Li *et al*. (*31*) extracted the essential oil of sea buckthorn by a combination of ultrasound and microwave-assisted extraction using ionic liquids as extraction solvents and compared it with conventional hydrodistillation. These authors were able to obtain (0.095±0.004) % essential oil by ionic liquid-based ultrasonic/microwave-assisted simultaneous distillation and extraction and (0.089±0.003) % by conventional hydrodistillation. Interestingly, these authors found that the extraction of the essential oil by microwave, ultrasound or conventional hydrodistillation contained the same volatile constituents. In contrast, in our study, we found that the essential oil extracted by modified hydrodistillation had a higher amount of individual compounds and better antioxidant ability than the oil extracted by conventional hydrodistillation. The higher recovery rate that we observed in this study confirms the effectiveness of Triton X-100 as a macerating agent. Another important reason for the higher recovery rates of the essential oil in this study than in the previous studies cited in Table 2 could be the controlled low temperature of the condenser (10 °C).

**Table 2:** The details of validation experiments for the maceration-mediated hydrodistillation of the essential oil from sea buckthorn berries

Treatment condition					Hydrodistillation parameter		*Y*(essential oil)/(g/100 g)
A: (*V*(Triton X-100)/*m*(sample))/(mL/100 g)	B: *w*(NaCl)/(g/100 g)	C: *t*/h			Temperature/°C	Condenser temperature/°C	
4.22	4.03	5.60			105	10	3.25
4.22	4.03	5.60			105	10	3.38
4.22	4.03	5.60			105	10	3.10
Experimental (mean±S.D.)							3.2±0.1
Predicted extraction (d≥0.05)							3.10
Conventional hydrodistillation		6.0			95	10	2.10
Increase in extraction efficiency							54.28 %
Literature report							
Cakir (*29*)			4.0	100		-	0.1 %
Yue *et al.* (*30*)			4.0	40		-	0.03 %
Li *et. al.* (*31*)			6.0	95		-	0.095 %

### Antioxidant properties of essential oil

**Fig. 2:**
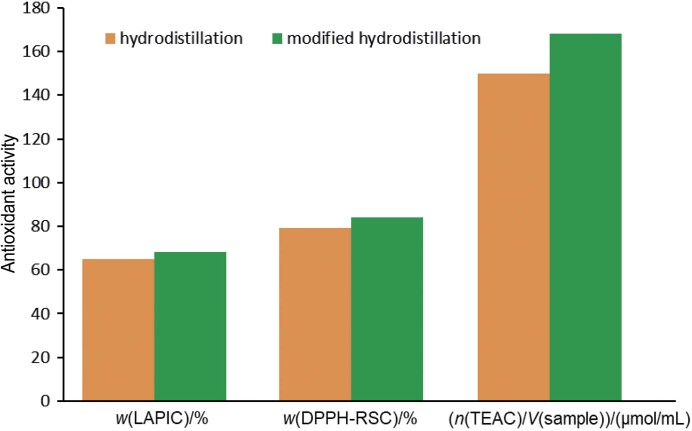
Comparison of antioxidant activities of the essential oil from sea buckthorn extracted by maceration-mediated hydrodistillation (modified) and conventional hydrodistillation. LAPIC=linoleic acid peroxidation inhibition capacity, FRSC=free radical scavenging capacity and TEAC=Trolox equivalent antioxidant capacity

The antioxidant activity of essential oil extracted by modified hydrodistillation and conventional hydrodistillation was determined in terms of its ability to scavenge free radicals, inhibit linoleic acid peroxidation and neutralise ABTS radical cations. The results of the antioxidant activity of the essential oil showed a significant difference between the two essential oils extracted by the modified hydrodistillation and the conventional method (Fig. 2). This difference in antioxidant activity could be attributed to the increase in the relative abundance of caryophyllene in the essential oil extracted with modified hydrodistillation. Similarly, TEAC is the most reliable assay to evaluate the antioxidant capacity of bioactive compounds based on their ability to reduce ABTS radical cations. The antioxidant activity of the extracts obtained by modified hydrodistillation and conventional hydrodistillation, expressed in Trolox equivalents, was 168 and 150 µmol/mL, respectively. The free radical scavenging ability of the essential oils obtained by the two hydrodistillation methods was also significantly different (p≤0.05). Moreover, the preservative potential of the extracted oil was evaluated based on the inhibition of linoleic acid peroxidation. The peroxide inhibition potential of the essential oil obtained by the modified and conventional hydrodistillation in linoleic acid varied up to 68.16 and 65.11 % respectively. Overall, the results show that the modified hydrodistillation yielded the essential oil with significantly higher antioxidant activities (free radical scavenging, inhibition of linoleic acid peroxidation and TEAC). The increase in the antioxidant ability of the essential oil obtained by modified hydrodistillation may be related to the hydrolytic/maceration potential of the surfactant (Triton X-100) and the thermodynamics of the water during modified hydrodistillation. Up to now, no maceration-mediated extraction of of the essential oil has been carried out. However, conventional hydrodistillation and solvent extraction showed the presence of potential bioactive antioxidant compounds in sea buckthorn berries (*3*).

### GC-MS characterisation

**Fig. 3:**
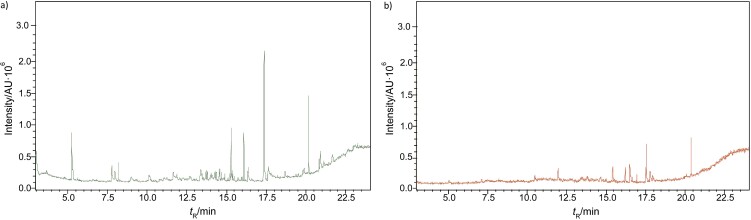
Gas chromatogram showing the compounds of the essential oil from sea buckthorn obtained using: a) maceration-mediated hydrodistillation and b) conventional hydrodistillation

Metabolic profiling of essential oil using gas chromatography coupled with mass spectrometry (GC-MS) showed that the essential oil can be extracted more effectively by modified hydrodistillation. Fig. 3 compares the relative abundance of different bioactive compounds in the essential oil obtained by modified hydrodistillation (Fig. 3a) and conventional hydrodistillation (Fig. 3b). Caryophyllene was the most abundant volatile terpene (37.25 %), followed by (Z)-9-octadecanoic acid methyl ester (12.74 %) and d-limonene (10.23 %) in the essential oil produced by modified hydrodistillation. The relative abundance of (Z)-9-octadecanoic acid methyl ester in the essential oil obtained by conventional hydrodistillation (20.70 %) was higher than that obtained by modified hydrodistillation, namely 12.74 %. Fig. 3 shows the presence of eight valuable aroma compounds in essential oil obtained by conventional hydrodistillation (control) and modified hydrodistillation (treated). The most abundant aroma compound detected in both the control and the treated essential oil was (Z)-9-octadecanoic acid methyl ester with a relative abundance of 20.70 and 23.74 % at a retention time of 16.68 and 20.035 min, respectively. The least abundant aroma compounds were 8,11-octadecadienoic acid methyl ester and oleic acid in both the control and treated sample. In addition, the essential oil obtained by either distillation may contain *n*-hexadecanoic acid, oleic acid, heptanal, nonanal and *n*-octanal (Table 3).

**Table 3:** Gas chromatography-mass spectrometry profile of the essential oil from sea buckthorn obtained under optimal conditions: a) maceration-modified hydrodistillation and b) conventional hydrodistillation

					Relative abundance/%	
Retention time	Compound name	Molecular formula	Molecular mass	Most probable structure	Hydrodistillation	
					conventional	maceration-mediated
5.735	Limonene	C_6_H_14_	72	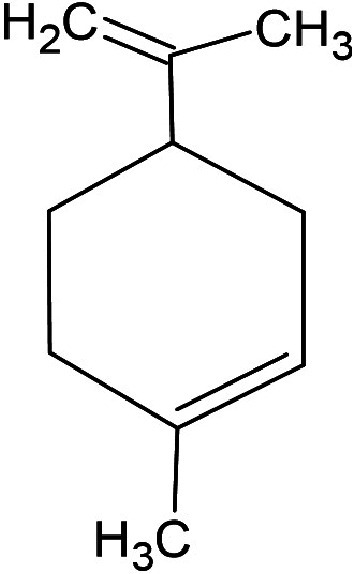	1.25	10.23
7.789	Heptanal	C_7_H_14_O	114	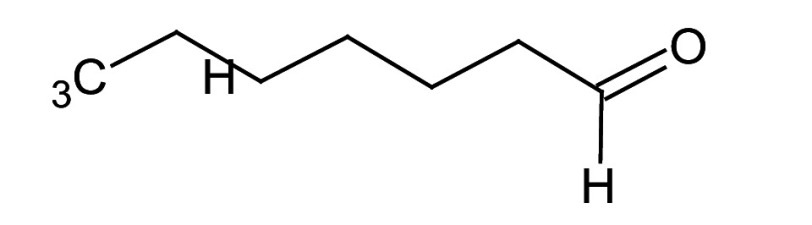	ND	1.46
8.710	Heptanoic acid methyl ester	C_8_H_16_O_2_	144	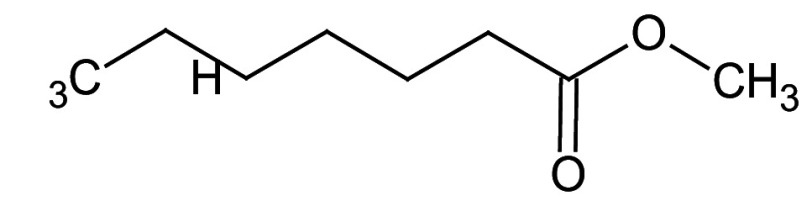	ND	3.92
9.218	Nonanal	C_9_H_18_O	142	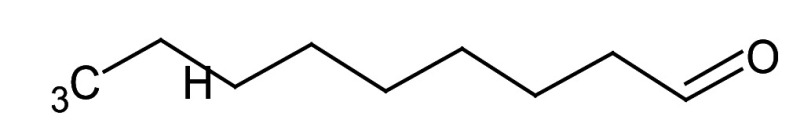	ND	3.18
10.420	*n*-Octanal	C_8_ H_16_O	97	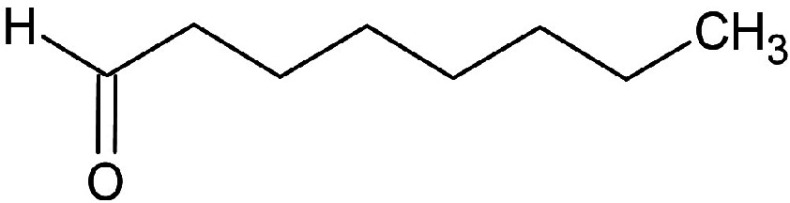	1.47	2.54
15.460	Hexadecanoic acid methyl ester	C_17_H_34_O_2_	270	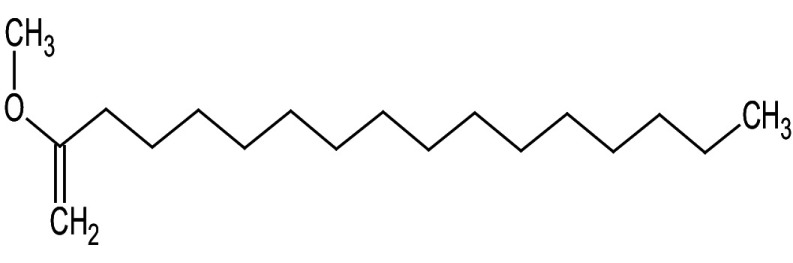	7.73	8.25
15.700	*n*-Hexadecanoic acid	C_16_H_32_O_2_	256	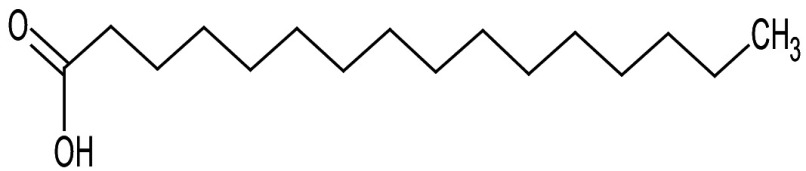	12.99	ND
16.649	8,11-Octadecadienoic acid methyl ester	C_19_H_34_O_2_	294	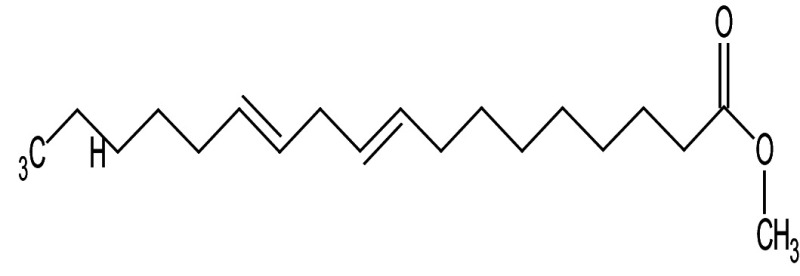	7.41	7.33
16.929	Oleic acid	C_18_H_34_O_2_	282	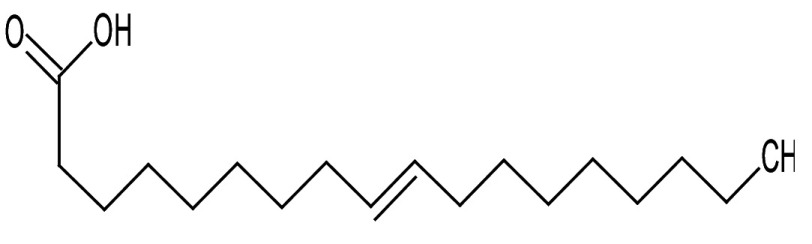	8.27	2.75
17.451	Caryophyllene	C_15_H_24_	204	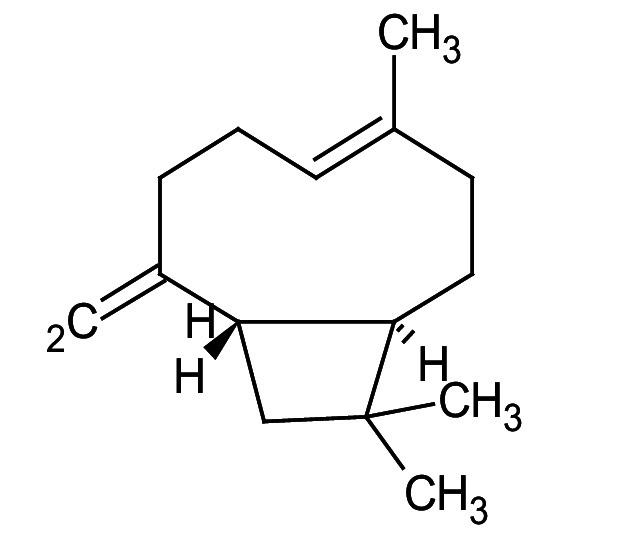	9.89	37.25
17.610	Isopropyl myristate	C_17_H_34_O_2_	270	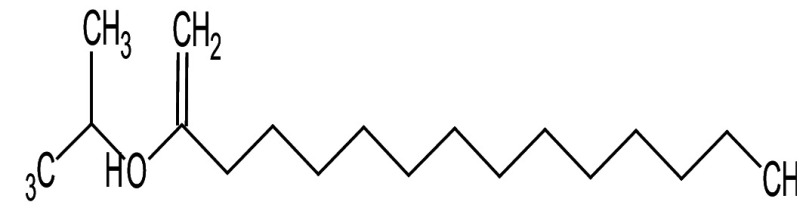	13.04	8.70
19.336	1,2-Benzenedicarboxylic acid, bis (2-methyl propyl) ester	C_16_H_22_O_4_	278	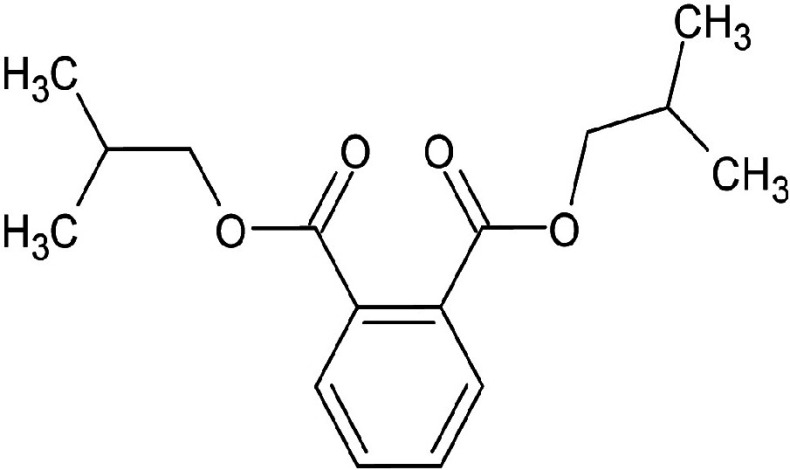	19.98	4.19
20.085	(Z)-9-Octadecanoic acid methyl ester	C_19_H_36_O_2_	296	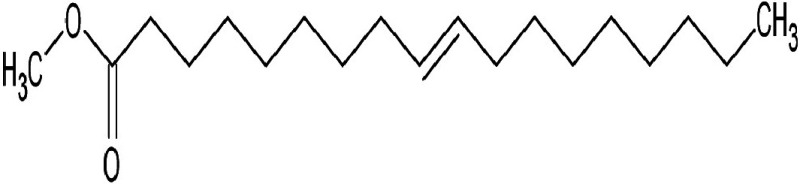	20.70	12.74

ND=not detected

A careful study of the literature shows that Cakir (*29*) extracted essential oil by steam distillation with ethyl decanoate as the main component at 39.4 %. Previously, Yue *et al.* (*30*) had obtained large amounts of *n*-hexadecanoic acid in all three extracted oils from sea buckthorn seeds, pulp and leaves at 36.64, 32.88 and 26.07 %, respectively, by conventional hydrodistillation. Li *et al.* (*31*) extracted myristic acid (10.24±0.15) and (Z)-8-dodecen-1-yl acetate (9.22±0.09) as the main components in essential oil by ionic liquid-based ultrasonic/microwave-assisted simultaneous distillation and extraction and conventional hydrodistillation, respectively. Recently, Sanwal *et al.* (*10*) reported methyl palmitate (25.85±0.01) as an abundant component in essential oil when ultrasound-assisted extraction was applied under optimal conditions. However, the technique we presented for extracting essential oil from sea buckthorn berries simultaneously improved the recovery of the essential oil and reduced the extraction time and associated costs. Moreover, the essential oil obtained by modified hydrodistillation was found to be rich in (−)-β-caryophyllene and other high-value bioactive compounds (Table 3).

## CONCLUSIONS

The essential oil of sea buckthorn can serve as a potential substitute for the food and pharmaceutical industries, as it contains bioactive compounds such as terpenes and short-chain fatty acid esters. However, many of these valuable volatiles are lost or degraded during extraction/distillation and, for similar reasons, the extraction of essential oil has remained a challenging task. This study concluded that modified hydrodistillation can work more efficiently compared to conventional hydrodistillation to extract essential oil. Modified hydrodistillation provided more than 54 % higher yield of essential oil, and this increase could be due to several known and unknown factors. Some of the former are: (*i*) better control of the extraction thermodynamics, and (ii) the addition of Triton X-100 (non-ionic surfactant) can be thought to improve mass transfer, while the presence of NaCl changes the thermodynamics of the water, which helps to capture the entrapped aroma. In contrast, the design of tube, especially the length and angle of the collector arm, could be responsible for increasing the yield of essential oil. In the present study, it was found that both the bending angle and the arm length of the Clevenger tube need to be further investigated. Conventional hydrodistillation was excluded due to limited recovery rates and it was found that this innovative technique contributes to the rapid release of volatile compounds by simultaneously altering the boiling point and surface tension of the extraction solvent. The extracted essential oil was rich in (−)-β-caryophyllene (37 %) and short-chain fatty acid esters.

## Appendix

**Table S1:** Analysis of variance (ANOVA) report for maceration-mediated hydrodistillation of the essential oil from sea buckthorn

Source	Sum of squares	df	Mean square	F-value	p-value
Model	54.03	9	6.00	261.47	<0.0001
A: Triton X-100	0.01	1	0.01	0.55	0.48
B: NaCl	0.23	1	0.23	10.07	0.0099
C: time	1.03	1	1.03	44.85	<0.0001
AB	0.05	1	0.05	1.96	0.19
AC	1.05	1	1.05	45.79	<0.0001
BC	0.05	1	0.05	1.96	0.19
A^2^	28.36	1	28.36	1235.27	<0.0001
B^2^	25.24	1	25.24	1099.15	<0.0001
C^2^	6.80	1	6.80	296.16	<0.0001
Residual	0.23	10	0.02		
Lack-of-fit	0.16	5	0.03	2.13	0.21
Pure error	0.07	5	0.015		
Cor total	54.26	19			
